# The Longitudinal Measurement of Reasoning Abilities in Students With Special Educational Needs

**DOI:** 10.3389/fpsyg.2019.00232

**Published:** 2019-02-11

**Authors:** Timo Gnambs, Lena Nusser

**Affiliations:** ^1^Educational Measurement, Leibniz Institute for Educational Trajectories, Bamberg, Germany; ^2^Institute for Education and Psychology, Johannes Kepler University Linz, Linz, Austria; ^3^Early Childhood and School Education, Leibniz Institute for Educational Trajectories, Bamberg, Germany

**Keywords:** reasoning, special educational needs, differential item functioning, educational large-scale assessment, longitudinal

## Abstract

Students with special educational needs in the area of learning (SEN-L) have learning disabilities that can lead to academic difficulties in regular schools. In Germany, these students are frequently enrolled in special schools providing specific training and support for these students. Because of their cognitive difficulties, it is unclear whether standard achievement tests that are typically administered in educational large-scale assessments (LSA) are suitable of students with SEN-L. The present study evaluated the psychometric properties of a short instrument for the assessment of reasoning abilities that was administered as part of a longitudinal LSA to German students from special schools (*N* = 324) and basic secondary schools (*N* = 338) twice within 6 years. Item response modeling demonstrated an essentially unidimensional scale for both school types. Few items exhibited systematic differential item functioning (DIF) between students with and without SEN-L, allowing for valid cross-group comparisons. However, change analyses across the two time points needed to account for longitudinal DIF among students with SEN-L. Overall, the cognitive test allowed for a valid measurement of reasoning abilities in students with SEN-L and comparative analyses regarding students without SEN-L. These results demonstrate the feasibility of incorporating students with SEN-L into educational LSAs.

## Introduction

Domain general cognitive abilities such as basic reasoning skills are a central predictor of many important life outcomes (e.g., Deary et al., 2007; Strenze, 2007; Gnambs, 2017). In the educational realm, they shape, for example, the development of domain-specific competences such as reading comprehension or mathematical skills and explain underachievement among risk groups (Weinert et al., 2011). Consequently, a valid and fair assessment of basic cognitive abilities is an essential prerequisite for the investigation of group differences in educational achievement or for the study of educational trajectories across students’ school careers. This poses a particular challenge in educational large-scale assessments (LSA) such as the *National Assessment of Educational Progress* in the United States (NAEP; e.g., National Center for Education Statistics, 2018) or the German *National Educational Panel Study* (NEPS; Blossfeld et al., 2011) that strive to include students from different, frequently rather heterogeneous, school tracks to reflect the entire educational system of a country. In these studies, the inclusion of disadvantaged students such as those with special educational needs (SEN; e.g., students with learning or language disabilities) can be difficult because these students exhibit highly variable cognitive abilities. Frequently, standard assessment instruments that are suitable for other students exhibit rather poor measurement properties for students with SEN (e.g., Südkamp et al., 2015; Pohl et al., 2016). Therefore, these students are either not included in LSAs at all or they are tested with specialized instruments, for example, including fewer items or allowing for a longer testing time (e.g., Pitoniak and Royer, 2001; Lutkus et al., 2004; for an overview see also Heydrich et al., 2013). Consequently, information on students with SEN is frequently not available in many LSAs or (because of the use of different instruments) not comparable to students from other school forms. Therefore, this study focuses on students with SEN in the area of learning that attended special schools with curricula specifically addressing these students’ educational needs and evaluated the feasibility of including these students in a longitudinal LSA. Particularly, we examined the measurement properties of a short instrument for the assessment of basic reasoning abilities, its measurement invariance with regard to students without SEN that attended basic secondary schools, and the change trajectories of reasoning abilities across a period of 6 years.

### Testing Students With Special Educational Needs

Students with SENs in the area of learning (SEN-L) are severely impaired in their ability to systematically acquire and retain new information which, consequently, interferes with their academic performance. Because the cognitive abilities of these students also fall below the normal range^1^, they typically have inferior grades and poor school leaving certificates. Thus, in regular schools these students cannot exhaust their academic potential, even when receiving additional educational support. In Germany, students with SEN-L are predominantly enrolled in special schools that provide special training and support for students with learning disabilities because they are unable to follow regular school lessons. Although inclusive education of students with SEN-L together with students that do not have SENs is becoming increasingly prevalent in Germany, most student with SEN-L attend these special schools (Dietze, 2012). In Germany, about 200,000 children and adolescents are diagnosed with SEN-L (Autorengruppe Bildungsbericht, 2018).

Students with SEN-L are described to have persistent and far-reaching limitations in coping with academic requirements (Klauer and Lauth, 1997) which is particularly evident in their insufficient ability to acquire cognitive-verbal and abstract content (Grünke and Grosche, 2014). Although these students are a rather heterogeneous group with a wide range of cognitive abilities as well as inter-individually different competence profiles (Müller et al., 2013), they exhibit substantially lower levels of cognitive abilities than regular students. Various studies have shown that students with SEN-L, on average, show lower performance in tests assessing, for example, reading competence (Pohl et al., 2016), mathematical competence (Wocken and Gröhlich, 2009), or reasoning abilities (Kocaj et al., 2014; Nusser and Messingschlager, 2018) as compared to students from regular schools. The poor performance of students with SEN-L in achievement tests can be attributed to various aspects, such as weaker language skills, slower learning performance, and reduced attention span (Grünke and Grosche, 2014). Since this group of students uses less effective strategies in learning processes, such as the organization of separate steps (Klauer and Lauth, 1997), difficulties may arise from superficial extraction of information, in particular when processing cognitive tasks (Scruggs et al., 1985; Hessels, 2009). Moreover, these students tend to have a less in-depth understanding of test instructions (Hessels-Schlatter, 2002), resulting in increased difficulties in following given guidelines, processing instructional hints, and applying them correctly to the presented tasks (Wong et al., 1982; Nusser and Weinert, 2017). In addition to the dispositional conditions of students with SEN-L, test characteristics may also have an influence on test performance. Reliability and validity may be impaired if items are not appropriate in terms of their difficulty for the target population (Nusser, 2018). Usually, target group-specific preconditions are not taken into account when developing diagnostic instruments. Thus, certain access skills, such as reading skills, attention span, and correct application of test instructions are neglected in this process (Renner and Mickley, 2015).

In light of these difficulties, a number of studies highlighted severe problems when administering instruments that were designed for students from regular schools to students with SEN (e.g., Bolt and Ysseldyke, 2008; Südkamp et al., 2015; Pohl et al., 2016). For example, Südkamp et al. (2015) were unable to identify an acceptable measurement model for a reading competence test that was administered to students with SEN-L in grade 5 of special schools, although the same test showed satisfactory results among students without SEN-L attending regular schools. Among students with SEN-L many items exhibited rather low item discriminations and severe misfit to the Rasch (1980) model that resulted in a substantially lower test reliability as compared to students from regular schools. Moreover, students with SEN-L exhibited a substantially larger amount of missing values, presumably because the test was too difficult for them. Taken together, the authors concluded that the reading competence test neither allowed for “substantive interpretations of the competence level of students with SEN-L” (p. 18) nor valid comparisons with students from regular schools. Similar results were observed by Bolt and Ysseldyke (2008) who identified severe differential item functioning on a mathematical test across different groups of students with SEN. Regarding tests of reasoning abilities, respective comparative studies in LSAs are still missing. However, there is evidence that for people with severe intellectual disabilities the factor structure of well-established intelligence batteries might break down (Jones et al., 2006; MacLean et al., 2011; for opposing results see Reynolds et al., 2013). This highlights that many cognitive measures that were originally developed for a general student population are either not comparable for students with SEN or, even worse, do not allow for a coherent construct measurement among students with SEN. More importantly, measurement invariance of many cognitive tests is rarely explicitly examined for test-takers with cognitive disabilities (see the review by Renner and Mickley, 2015). Therefore, cognitive testing of students with SEN-L frequently relies on psychological instruments without knowing whether their measurement properties hold in this specific subgroup.

### The Present Study

Educational large-scale assessments typically administer identical instruments to students from different school tracks. Unless these tests function comparable for all students and measure identical constructs, group comparisons (e.g., between different school types) or changes across measurement occasions (e.g., to study cognitive development) cannot be properly evaluated. Given the outlined difficulties of students with SEN-L, it is unclear whether achievement tests that were originally developed for students without SEN-L are suitable for administration in special schools. Therefore, we evaluated a short instrument for the measurement of basic reasoning abilities that is routinely administered in a German longitudinal large-scale assessment, the NEPS (Blossfeld et al., 2011). The test properties are compared between students with SEN-L that attend special schools and students without SEN-L from basic secondary schools regarding its internal validity and test fairness for cross-sectional comparisons across school types and for longitudinal comparisons across 6 years.

## Materials and Methods

### Sample and Procedure

The National Educational Panel Study (NEPS) includes a representative sample of German students from lower secondary education in grade 9. The stratified, two-stage sampling procedure (for details see Steinhauer et al., 2015) acknowledged all major school types in Germany including, for example, schools leading to upper secondary education and university entrance qualification (*Gymnasium*), schools for basic secondary education (*Hauptschule*), and intermediate secondary schools (*Realschule*). Additionally, the NEPS also comprised participants attending special schools for students with SEN-L (*Förderschule*). The present study focuses on two measurement waves that administered identical tests in 2011 (grade 9) and 6 years later in 2017 to two subsamples of students from special schools and basic secondary schools. At the first measurement wave, responses from 1,085 students in special schools and from 3,388 students in basic secondary schools were available (see Nusser and Messingschlager, 2018). For the second wave, all students from special schools that had consented to further psychological testing were eligible to participate (*N* = 680). In addition, a random comparison sample from former basic secondary school students was selected (*N* = 703) that was matched on the ability distribution of the special school students^2^. For this purpose, a proportional sampling procedure was applied: In the first step, different ability groups were created for both student groups. Then, for each proficiency group a random sample of students from regular school was drawn to match the sample size of the respective proficiency group among students from special schools. Thus, by design the two subsamples in the second wave exhibited similar reasoning abilities on the previous wave. In the end, valid responses from *N* = 324 former students of special schools and *N* = 338 former students from basic secondary schools were available at the second measurement wave (see Table 1). For this study, we focus on these 662 participants (45% women). In 2011, students were tested in small groups at their respective schools, whereas the follow-up assessment in 2017 was conducted in the participants’ private homes, where they were individually tested by experienced test administrators from a professional survey institute. Details on the data collection process including the test execution, the selection of test administrators, and the tracking of respondents are documented in the field reports provided on the project website^3^.

**Table 1:** Sample sizes and non-response for subsamples.

	Special schools	Secondary schools
Wave 1 (Grade 9)		
Participated	1,085	3,388
Wave 2 (Young adults)		
Field sample	680 (100%)	703 (100%)
Non-response:		
- Not part of target sample (e.g., deceased)	0 (0%)	2 (0%)
- Not reached (e.g., new address unknown)	160 (24%)	118 (17%)
- Unable to be tested (e.g., illness)	1 (0%)	1 (0%)
- Refused participation in entire study	105 (15%)	127 (18%)
- Refused participation in specific test	3 (0%)	17 (2%)
- Other (e.g., not available during field time)	87 (13%)	100 (14%)
Participated	324 (48%)	338 (49%)
Participated in Waves 1 and 2	279 (86%)	338 (100%)
Participated in Wave 2 only	45 (14%)	0 (0%)


### Ethics Statement

The Federal Ministries of Education in Germany approved the study. Ethical standards were approved by the NEPS. Written informed consent was given by the students and their parents in accordance with the Declaration of Helsinki. Moreover, informed consent was also given by the educational institutions to take part in the study. The consent procedure was approved by a special data protection and security officer of the NEPS. Students (as well as all other parties) could abort their participation at any time in the study. Further approval by an ethics committee was not required according to the local and national guidelines.

### Instrument

**FIGURE 1:**
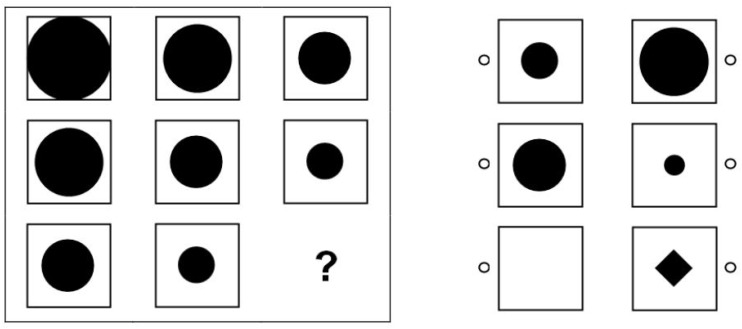
Example item of the NEPS reasoning test (Copyright Leibniz Institute for Educational Trajectories. Reproduced with permission).

Reasoning abilities were measured with a matrices test following Raven (1977) that included 12 items. The test was specifically constructed for administration in the NEPS (see Lang et al., 2014) to provide an economic assessment of participants’ general cognitive functioning. Thus, the test was not constructed for a comprehensive assessment of an individual’s intellectual capacities but aimed at providing a short indicator of basic reasoning skills for population level research. Each item consisted of one blank field and a number of fields containing geometrical elements that followed various logical rules (see Figure 1). Participants had to identify the underlying rules to insert the correct element into the blank field from a series of available response options. The items were distributed among three pages with four items each. The time limit for each page was 3 min. The available time was long enough to ensure that the test measured maximal performance rather than speed (Lang et al., 2014). Before responding to the first item, all respondents received a standardized instruction and a practice item. The total testing time was 11 min. The instrument exhibited satisfactory convergent validities with regard to Raven’s (1977) *Standard Progressive Matrices* in several samples of students and adults (*r*s between 0.44 and 0.61) and discriminant validity with regard to measures of crystallized intelligence, memory, and perceptual speed (see Lang et al., 2014). In the school setting (Wave 1), the test was presented in a paper-based booklet and the timing of the different parts of the test was supervised by the test administrator. In contrast, in the individual setting (Wave 2), the entire test (including the instruction and timing) was administered on the computer. Although a test administrator was present, she or he only interfered if the respondent indicated problems with the test. Previous studies showed that different test media (i.e., paper-based versus computer-based) are a negligible source of individual differences in cognitive ability measures such as the one administered in this study (Williams and McCord, 2006; Schroeders and Wilhelm, 2010). Therefore, it is unlikely that mode effects substantially biased the comparison of reasoning abilities over time. At both measurement occasions the reasoning test was administered first before working on other cognitive tests.

### Statistical Analyses

The measurement structure of the reasoning test administered to the two subsamples was examined by fitting a unidimensional Rasch (1980) model with Gauss-Hermite quadrature (21 nodes) to the responses of each measurement wave. In line with prevalent recommendations, missing responses were ignored during model estimation (Pohl et al., 2014). Model fit was evaluated using the weighted mean square (WMNSQ) statistic that has an expectation of 1. Fit statistics greater than 1 indicate more variation (or noise) than predicted by the Rasch model, that is, an underfit with the model. Following prevalent rules of the thumbs (e.g., Smith et al., 2008; Pohl and Carstensen, 2013), we considered values of WMNSQ < 1.15 as indicative of close item fit, 1.15 ≤ WMNSQ < 1.20 as small item misfit, and WMNSQ ≥ 1.20 as considerable item misfit. Because the *t*-statistic associated with the WMNSQ is highly sample dependent (Karabatsos, 2000; Linacre, 2003), we put less emphasis on the inference test. The local independence assumption of the Rasch model was evaluated by inspecting the model residuals. Approximately zero-order correlations as indicated by Yen’s (1984) *Q*_3_ indicate essential unidimensionality. Because in case of locally independent items, the *Q*_3_ statistic tends to be slightly negative, we report the corrected *Q*_3_ that has an expected value of 0. Following prevalent practice (e.g., Yen, 1993; Amtmann et al., 2010), values of *Q*_3_ falling below 0.20 indicate essential unidimensionality. Overall judgment of the fit of an item was based on all fit indicators and a visual inspection of the respective item characteristic curve.

In sensitivity analyses we compared the Rasch (1980) model to a two-parametric logistic test model (Birnbaum, 1968) that allowed for different item discriminations. Model comparisons were conducted using the Bayesian Information Criterion (BIC; Schwartz, 1978) that indicates a better fit at lower values. Moreover, the strength of evidence in favor of a model was quantified using the Bayes Factor (BF; see Wagenmakers, 2007). Following Raftery (1995), we considered BFs between 1 and 3 as weak evidence, BFs between 3 and 20 as positive evidence, BFs between 20 and 150 as strong evidence, and BFs exceeding 150 as very strong evidence in favor of a model.

The reasoning test should measure the same construct for students in special and basic secondary schools across the two measurement occasions. If some items favored certain subgroups, a comparison of ability scores between these subgroups would be biased and, thus, unfair. Differential item functioning (DIF) refers to differences in item difficulties (uniform DIF) or differences in item discriminations (non-uniform DIF). Because the Rasch model assumes identical item discriminations we focused on the former. Uniform DIF between school types was examined using a multi-group Rasch model, in which main effects of the two subgroups as well as differential effects of the subgroups on item difficulty were modeled. Following the Educational Testing Service (Holland and Wainer, 1993), we considered standardized differences in item difficulties up to Cohen’s *d* = 0.25 (i.e., a quarter of a standard deviation) as negligible DIF. A modified version of Raju’s (1988; 1990) significance test that computes the area between the item characteristic curves for the two groups was used to test for the presence of non-negligible DIF. Following Murphy and Myors (1999), we adopted a minimum effect hypothesis and tested whether the difference in item difficulties was significantly (α = 0.05) greater than our threshold for non-negligible DIF, that is, *d* = 0.25 (see Fischer et al., 2016). Additionally, test fairness was examined by comparing the fit of a model including DIF to a model that only included main effects and no DIF. Longitudinal DIF across the two measurement occasions was evaluated by specifying a two-dimensional Rasch model. Again, DIF was evaluated using a modified version of Raju’s (1988; 1990) signed area test with a minimum effect hypothesis (Fischer et al., 2016). The analyses were conducted in *R* version 3.5.1 (R Core Team, 2018) using the *TAM* package version 2.12-18 (Robitzsch et al., 2018).

## Results

The response rates at Wave 2 in the two subsamples from special schools and basic secondary schools fell at 48 and 49%, respectively (see Table 1), and did not differ significantly [χ^2^(1) = 0.00, *p* = 0.962, *r*_φ_ = 0.00]. Although the reachability and motivation of the target group was somewhat low, the response rates were comparable between both school types. The two groups were also highly comparable regarding central socio-demographic characteristics (see Table 2). Although students from basic secondary schools included slightly more participants with migration background, the difference was rather small (*r*_φ_ = 0.13, *p* < 0.001). Due to the matching procedure, the two groups did not differ with regard to their reasoning abilities at the first measurement occasion (*d* = -0.09, *t*_Welch_(597.43) = -1.49, *p* = 0.136).

**Table 2:** Sample descriptions.

	Special schools	Secondary schools	
*N*	324	338	
Percent women	46%	45%	χ^2^(1) = 0.07,
			*p* = 0.794, *r*_φ_ = 0.01
Mean age (*SD*)	21.95 (0.59)	21.84 (0.69)	*t*_Welch_(651.31) = 2.05
Age range	[21, 24]	[21, 25]	*p* = 0.041, *d* = 0.11
Percent migration background	15%	26%	χ^2^(1) = 11.31,
			*p* < 0.001, *r*_φ_ = 0.13
Mean reasoning at wave 1 (*SD*)^1^	5.00 (2.52)	5.31 (2.57)	*t*_Welch_(597.43) = -1.49
			*p* = 0.136, *d* = -0.09


*Results refer to the sample at the second measurement occasion. Reasoning was quantified as number correct scores.*

### Distributions of Missing Values

**FIGURE 2:**
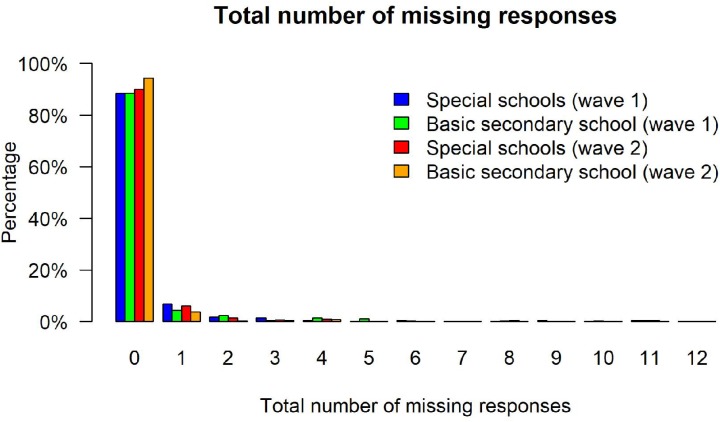
Total number of missing responses.

Achievement tests can exhibit different kinds of missing values resulting, for example, from omitted items or items that test takers did not reach. In the present sample, the test takers skipped rather few items. Among students from special schools, respondents had, on average, *M* = 0.08 (*SD* = 0.37) and *M* = 0.16 (*SD* = 0.64) omitted items at the first and second measurement occasion, whereas the respective frequencies were *M* = 0.08 (*SD* = 0.43) and *M* = 0.09 (*SD* = 0.44) for students from basic secondary schools. Importantly, missing rates did not differ significantly between the two groups, neither at the first measurement occasion, *t*_Welch_(614.62) = -0.08, *p* = 0.937, *d* = -0.00, nor the second measurement occasion, *t*_Welch_(570.94) = -1.68, *p* = 0.094, *d* = -0.09. Similar, we observed few missing values from items that were not reached because respondents ran out of time or lacked motivation and, thus, aborted the test before all items were administered. Among students from special schools about *M* = 0.08 (*SD* = 0.73) and *M* = 0.05 (*SD* = 0.52) items were not reached at the two measurement occasions, whereas the respective frequencies amounted to *M* = 0.09 (*SD* = 0.76) and *M* = 0.01 (*SD* = 0.16) for students from basic secondary schools. Again, missing rates did not differ significantly between the two groups, *t*_Welch_(600.77) = 0.21, *p* = 0.831, *d* = 0.01 and *t*_Welch_(382.93) = -1.23, *p* = 0.219, *d* = -0.07. Consequently, there was no noticeable difference in the total amount of missing values between the school types (see Figure 2). Between 88% and 94% of the respondents had no missing values at all in the different subsamples, whereas 4–7% had a single missing value. Overall, these results indicate that the two groups did not produce different missing rates. Finally, we also examined missing responses per item in order to evaluate whether specific items functioned differently. However, all items exhibited negligible missing rates between 0% and 5% (see Table 3). The highest missing rate was observed for the fourth item (between 3% to 5%) that was presented last on the first page of the test. Thus, some respondents seemed to have misjudged the available time and omitted the respective item. Again, no differences between special schools and basic secondary schools in the missing rates for this item were observed, neither in Wave 1 [χ^2^(1) = 0.40, *p* = 0.529, *r*_φ_ = 0.03] nor in Wave 2 [χ^2^(1) = 2.28, *p* = 0.131, *r*_φ_ = 0.06].

**Table 3:** Percentage of missing values by item.

	Special schools								Secondary schools							
		
	Wave 1				Wave 2				Wave 1				Wave 2			
												
Item	*N*	NR %	OM %	TO %	*N*	NR %	OM %	TO %	*N*	NR %	OM %	TO %	*N*	NR %	OM %	TO %
1	275	0.00	0.36	1.43	320	0.00	1.23	1.23	320	0.00	0.89	5.33	336	0.00	0.59	0.59
2	272	0.36	0.00	2.51	320	0.00	1.23	1.23	323	0.00	0.00	4.44	335	0.00	0.89	0.89
3	273	0.36	1.43	2.15	319	0.00	1.54	1.54	327	0.00	0.59	3.25	334	0.00	1.18	1.18
4	266	0.36	2.51	4.66	307	0.00	5.25	5.25	323	0.59	1.48	4.44	329	0.00	2.66	2.66
5	276	0.36	0.00	1.08	322	0.31	0.31	0.62	332	0.59	0.59	1.78	338	0.00	0.00	0.00
6	275	0.36	0.72	1.43	318	0.31	1.54	1.85	325	0.59	1.48	3.85	335	0.00	0.89	0.89
7	272	0.36	1.08	2.51	321	0.31	0.62	0.93	327	0.59	1.18	3.25	336	0.00	0.59	0.59
8	271	0.36	1.79	2.87	320	0.31	0.93	1.23	332	0.59	0.89	1.78	336	0.00	0.59	0.59
9	275	0.72	0.36	1.43	320	0.62	0.62	1.23	332	0.89	0.00	1.78	338	0.00	0.00	0.00
10	276	0.72	0.00	1.08	318	0.93	0.93	1.85	332	1.18	0.59	1.78	335	0.30	0.59	0.89
11	274	1.79	0.00	1.79	320	0.93	0.31	1.23	331	1.78	0.30	2.07	336	0.30	0.30	0.59
12	272	2.15	0.00	2.51	316	0.93	1.54	2.47	329	2.37	0.00	2.66	335	0.30	0.59	0.89


**N*, number of valid responses; NR, percentage of items not reached; OM, percentage of omitted items; TO, total percentage of missing values.*

### Unidimensional Rasch Scaling

Among students from special schools, the percentage of correct responses varied between 17% and 67% (*Mdn* = 43%) and between 31% and 86% (*Mdn* = 54%) at the two measurement occasions. The respective results for students from basic secondary schools were *Mdn* = 46% (*Min* = 26%, *Max* = 78%) and *Mdn* = 66% (*Min* = 38%, *Max* = 83%). Thus, the items covered a broad range of the ability spectrum. Because there were some missing responses, these probabilities cannot be straightforwardly interpreted as an index of item difficulty. Therefore, we fitted a unidimensional Rasch (1980) model to the responses in each subgroup. The respective results are summarized in Tables 4, 5. The item difficulties were estimated by constraining the mean of the ability distribution to 0. At the first measurement occasion, the estimated item difficulties ranged from -1.48 to 1.85 (*Mdn* = 0.36) among students in special school and from -1.28 to 1.22 (*Mdn* = 0.20) among students in basis secondary school. The respective estimates at the second measurement occasion were *Mdn* = -0.18 (*Min* = -2.05, *Max* = 0.92) and *Mdn* = -0.76 (*Min* = -1.82, *Max* = 0.59), respectively. For students with SENs, the fit of the items to the Rasch model can be considered good at both measurement occasions: values of the WMNSQ ranged from 0.90 to 1.08 and no *t*-value indicated a significant (α = 0.05) model violation. Moreover, a visual inspection of the item characteristic curves (ICC) showed a close fit of the empirical and the model implied response distributions. Among secondary school students, item 3 exhibited a minor misfit (WMNSQ = 1.16/1.18, *t* = 2.92/3.89). The empirical ICC was somewhat flatter than expected by the Rasch model. However, for the remaining items there was no indication of severe item over- or underfit. Moreover, there were negligible residual correlations as indicated by the *Q*_3_ statistic that did not exceed 0.20 for any item. Overall, the Rasch model showed a satisfactory fit in both school types and at both measurement occasions.

**Table 4:** Item parameters for Students from special schools.

	Wave 1								Wave 2							
		
Item	*N*	Perc. correct %	ξ	*SE*_ξ_	WMNSQ	*t*	*r_it_*	*Q*_3_	*N*	Perc. Correct %	ξ	*SE*_ξ_	WMNSQ	*t*	*r_it_*	*Q*_3_
1	275	67	-0.87	0.14	0.94	-0.94	0.38	0.07	320	65	-0.71	0.13	1.00	0.00	0.29	0.04
2	272	78	-1.48	0.16	0.90	-1.26	0.41	0.09	320	86	-2.05	0.17	0.96	-0.38	0.31	0.07
3	273	21	1.56	0.16	1.06	0.69	0.19	0.06	319	45	0.22	0.12	1.08	1.85	0.17	0.05
4	266	17	1.85	0.17	1.06	0.57	0.18	0.04	307	34	0.77	0.13	1.07	1.36	0.15	0.07
5	276	42	0.38	0.13	1.06	1.18	0.26	0.07	322	55	-0.23	0.12	1.04	0.88	0.23	0.04
6	275	61	-0.53	0.14	0.90	-1.89	0.46	0.07	318	61	-0.50	0.12	0.94	-1.30	0.39	0.07
7	272	26	1.27	0.15	1.02	0.29	0.26	0.06	321	31	0.92	0.13	1.03	0.57	0.22	0.06
8	271	21	1.61	0.16	1.07	0.81	0.16	0.05	320	37	0.62	0.12	1.04	0.78	0.23	0.04
9	275	60	-0.49	0.14	0.97	-0.48	0.35	0.04	320	79	-1.53	0.15	0.95	-0.64	0.35	0.08
10	276	55	-0.24	0.13	0.95	-1.02	0.40	0.08	318	66	-0.76	0.13	0.92	-1.51	0.41	0.07
11	274	43	0.35	0.13	1.03	0.53	0.30	0.05	320	53	-0.13	0.12	1.03	0.66	0.26	0.07
12	272	19	1.69	0.16	1.00	0.03	0.27	0.05	316	34	0.58	0.12	0.93	-1.47	0.40	0.04


**N*, number of valid responses; Perc. correct, percentage of correct responses; ξ, item difficulty parameter; *SE*_ξ_, standard error of ξ; WMNSQ, weighted mean square error; *t*, *t*-value for WMSNQ; *r_*it*_*, item-total correlation; *Q*_*3*_, average adjusted Yen’s *Q*_*3*_ across all item pairs.*

**Table 5:** Item parameters for Students from basic secondary schools.

	Wave 1								Wave 2							
		
Item	*N*	Perc. Correct %	ξ	*SE*_ξ_	WMNSQ	*t*	*r_it_*	*Q*_3_	*N*	Perc. correct %	ξ	*SE*_ξ_	WMNSQ	*t*	*r_it_*	*Q*_3_
1	320	62	-0.54	0.12	0.98	-0.56	0.31	0.05	336	72	-1.12	0.13	1.03	0.46	0.26	0.06
2	323	76	-1.28	0.14	0.99	-0.14	0.26	0.09	335	83	-1.82	0.15	1.02	0.20	0.24	0.07
3	327	34	0.79	0.13	1.16	2.92	0.01	0.06	334	54	-0.18	0.12	1.18	3.89	0.08	0.07
4	323	30	0.98	0.13	1.04	0.76	0.17	0.04	329	50	0.02	0.12	1.04	1.04	0.25	0.06
5	332	50	-0.01	0.12	1.05	1.23	0.22	0.05	338	76	-1.34	0.14	0.98	-0.30	0.32	0.05
6	325	50	0.01	0.12	0.92	-2.00	0.41	0.07	335	65	-0.71	0.12	0.94	-1.32	0.42	0.07
7	327	27	1.22	0.13	1.01	0.16	0.24	0.04	336	38	0.59	0.12	1.02	0.34	0.28	0.06
8	332	35	0.73	0.12	0.97	-0.52	0.31	0.05	336	60	-0.46	0.12	1.02	0.40	0.29	0.05
9	332	67	-0.82	0.12	0.95	-0.99	0.36	0.08	338	82	-1.77	0.15	0.89	-1.28	0.45	0.07
10	332	51	-0.05	0.12	0.95	-1.37	0.36	0.07	335	69	-0.95	0.13	0.94	-1.10	0.40	0.07
11	331	41	0.40	0.12	1.01	0.13	0.28	0.06	336	67	-0.82	0.13	0.99	-0.17	0.32	0.04
12	329	27	1.15	0.13	0.97	-0.39	0.30	0.03	335	54	-0.19	0.12	0.98	-0.56	0.36	0.05


**N*, number of valid responses; Perc. Correct, percentage of correct responses; ξ, Item difficulty parameter; *SE*_ξ_, standard error of ξ; WMNSQ, weighted mean square error; *t*, *t*-value for WMSNQ; *r_*it*_*, item-total correlation; *Q*_*3*_, average adjusted Yen’s *Q*_*3*_ across all item pairs.*

As a sensitivity analysis, we also fitted a two-parametric logistic model (Birnbaum, 1968) to the responses in each subgroup that allowed for different item discrimination parameters. In line with the previous results, the BIC favored the more restrictive Rasch model in all cases (see Table 6). The BF for these model comparisons indicated positive to strong evidence for the Rasch model among students from regular schools and very strong evidence for the Rasch model among students from special schools (Raftery, 1995).

**Table 6:** Model comparisons.

	Rasch model			Two-parametric model			
			
	Deviance	Parameters	BIC	Deviance	Parameters	BIC	Bayes Factor
*Regular schools*							
First measurement	4,837	13	4,913	4,782	24	4,922	98
Second measurement	4,715	13	4,790	4,655	24	4,794	8
*Special schools*							
First measurement	3,679	13	3,753	3,633	24	3,768	1,996
Second measurement	4,602	13	4,677	4,556	24	4,694	7,131


*Parameters, number of estimated model parameters. BIC, Bayesian Information Criterion (Schwartz, 1978).*

**FIGURE 3:**
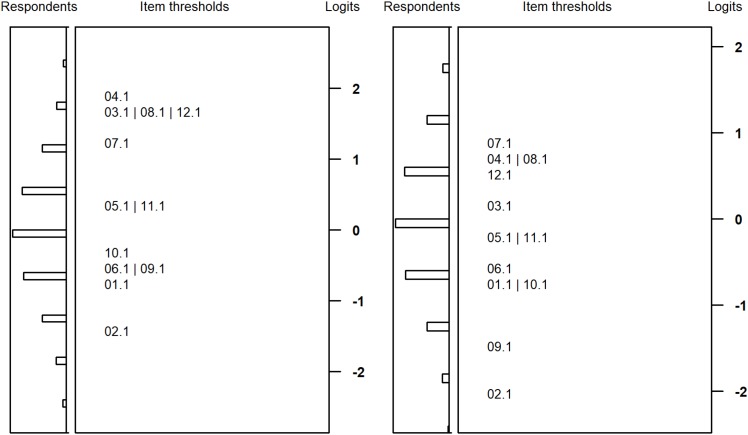
Wright maps for students with special educational needs at the first **(left)** and second measurement **(right)** occasion.

Figure 3, 4 plot the item difficulties of the reasoning items (left part of the plot) from the Rasch model and the ability of the test takers (right part) on the same scale. The mean of the ability distribution was constrained to zero for identification. The variance was estimated as 1.00 and 0.81 for students with SEN-L and as 0.70 and 0.76 for secondary school students. Thus, the test was better able to differentiate between respondents among students with SEN-L as compared to secondary school students. Although the items covered a wide range of the ability distribution, there were few items covering the upper peripheral ability area, particularly for students participating at the second measurement occasion. As a consequence, the test measured low and medium person abilities more precisely, whereas higher ability estimates exhibited larger standard errors of measurement. As expected, the marginal reliabilities (Adams, 2005) of the test were somewhat low but did not differ substantially between the two groups: 0.66/0.61 and 0.63/0.63 for students from special schools and secondary schools, respectively.

**FIGURE 4:**
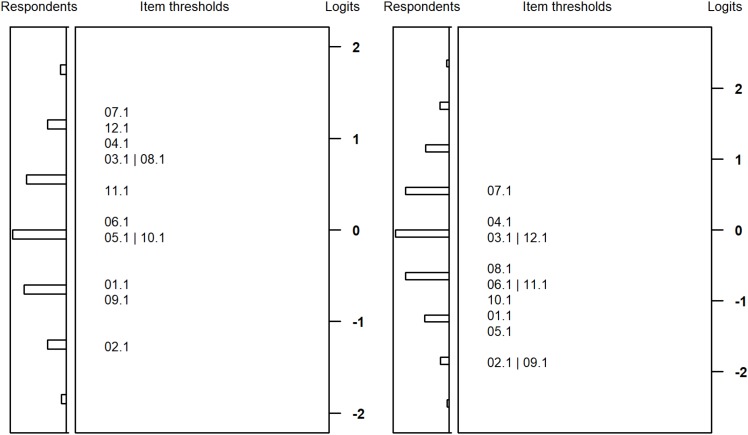
Wright maps for students from basic secondary schools at the first **(left)** and second measurement **(right)** occasion.

### Differential Item Functioning

Comparisons between students from different school types require comparable measurement structures of the reasoning test in both groups. Therefore, DIF was evaluated at each measurement occasion by specifying a unidimensional multi-group Rasch model and comparing the estimated item difficulties between the two groups. As summarized in Table 7, in Wave 1 four items resulted in different item parameters that were significantly larger than *d* = 0.25. However, the direction of the respective DIF effects were not consistent: whereas two items were significantly more difficult for students with SENs, the remaining two items were significantly easier. Moreover, the identified DIF did not have a pronounced impact on mean-level comparisons between the two school types. The multi-group model with free item parameters in each group resulted in a highly similar main effect for the school type (*d* = -0.22) as compared to a model that constrained the item parameters across groups (*d* = -0.19). Finally, model comparisons using the BIC favored the more restrictive model without DIF (BIC = 8,672.01) as compared to the more complex model with DIF effects (BIC = 8,680.25). The BF of 62 indicated strong evidence in favor of the model without DIF effects. Highly similar results were observed at the second measurement occasion (see Table 7). Although two items exhibited significant DIF, the direction of the respective effects were neither consistent nor did they affect mean level comparisons. Again, model comparisons favored the model without DIF (BIC = 9,455.89) as compared to a model with DIF effects (BIC = 9,479.28). The BF exceed 150 and, thus, indicated very strong evidence in favor of the more restrictive model without DIF. Together, these results do not indicate systematic and substantial DIF that might distort mean-level comparisons between students from special schools and students from basic secondary schools.

**Table 7:** Differential item functioning.

	Special vs. secondary schools		Wave 1 vs. wave 2	
		
Item	Wave 1	Wave 2	Special schools	Secondary Schools
1	-0.50 (-0.55*)	-0.10 (-0.11)	-0.81 (-0.85*)	-0.37 (-0.41)
2	-0.36 (-0.40)	-0.75 (-0.83*)	-0.08 (-0.09)	-0.40 (-0.45)
3	0.53 (0.59*)	-0.09 (-0.10)	0.67 (0.71)	0.02 (0.03)
4	0.63 (0.69*)	0.27 (0.30)	0.43 (0.45)	0.01 (0.01)
5	0.18 (0.20)	0.61 (0.67*)	-0.05 (-0.05)	0.39 (0.44)
6	-0.72 (-0.80*)	-0.29 (-0.32)	-0.68 (-0.73*)	-0.23 (-0.26)
7	-0.19 (-0.21)	-0.14 (-0.16)	-0.31 (-0.33)	-0.31 (-0.35)
8	0.63 (0.70*)	0.59 (0.65*)	0.33 (0.35)	0.25 (0.28)
9	0.15 (0.17)	-0.27 (-0.30)	0.38 (0.40)	0.01 (0.01)
10	-0.39 (-0.43)	-0.31 (-0.34)	-0.15 (-0.16)	-0.04 (-0.04)
11	-0.26 (-0.28)	0.20 (0.22)	-0.18 (-0.19)	0.27 (0.30)
12	-0.29 (-0.32)	-0.29 (-0.31)	0.45 (0.48)	0.40 (0.45)
Main effect with DIF	-0.20 (-0.22)	-0.49 (-0.54)		
Main effect without DIF	-0.17 (-0.19)	-0.53 (-0.58)	0.65 (0.70)	0.96 (1.08)


*Unstandardized differences in item parameters (with standardized differences in parentheses). ^∗^Difference significantly (*p* < 0.05) greater than 0.25.*

Finally, we also examined DIF across measurement occasions for each subgroup to evaluate whether longitudinal trajectories in reasoning abilities can be studied. To this end, we estimated a two-dimensional Rasch model (either for students from special schools or students from regular schools) that specified a latent factor for each measurement wave. The differences in item parameters and the results for the respective inference tests are summarized in Table 7. To compare the item parameters, we used a sum null constraint on the item parameters for each latent factor. For students with SEN-L, two items exhibited significant DIF exceeding *d* = 0.25. Both items were more difficult at the second measurement occasion. To evaluate whether the DIF biased longitudinal mean-level comparisons we compared two models: a model without DIF that constrained all item parameters over time and a model with DIF that estimated independent item parameters for the two DIF items and constrained the remaining item parameters over time. The model without DIF estimated a longitudinal change in reasoning for students with SEN-L of Cohen’s *d* = 0.70, whereas the respective effect was *d* = 0.88 for the model with partial invariance constraints. Thus, ignoring DIF for the two items introduced a small bias in the estimated mean-level change. Moreover, the information criteria also suggested that the model with partial measurement invariance (BIC = 8,338.18) provided a superior fit as compared to the model without DIF effects (BIC = 8,361.84). The BF exceeded 150 and, thus, indicated very strong evidence in favor of the partial invariance model. In contrast, for students from basic secondary school no items with significant longitudinal DIF were identified (see Table 7).

### Number Correct Scoring

Frequently researchers prefer to work with simple sum scores as compared to more complex latent variables. Therefore, we examined to what degree group comparisons using the number correct scores mirrored the respective latent variable analyses. Number correct scoring resulted in standardized mean differences between students from special schools and students from secondary schools of Cohen’s *d* = -0.08 and *d* = -0.34 at the two measurement occasions. In contrast, the Rasch model estimated differences of *d* = -0.19 and *d* = -0.58, respectively. Thus, using the more appropriate latent variable models that accounted for missing values and potential DIF resulted in substantially larger effects as compared to observed score analyses. Similar, the estimated change trajectories using the number correct scores were *d* = 0.59 for students with SEN-L and *d* = 0.90 for students from basic secondary schools, whereas Rasch modeling resulted in latent change trajectories of *d* = 0.88 and *d* = 1.08, respectively. Moreover, number correct scores resulted in rank-order stabilities between the two time points of *r* = 0.49 and *r* = 0.37 for students with and without SEN-L. In contrast, the longitudinal Rasch models estimated substantially larger retest correlations of *r* = 0.75 and *r* = 0.59, respectively.

## Discussion

The valid assessment of cognitive abilities of students with SEN-L poses an ongoing challenge for test developers, particularly in LSAs that administer identical instruments to heterogeneous school populations (Heydrich et al., 2013; Pohl et al., 2016). Therefore, the present study examined the longitudinal assessment of reasoning abilities in a sample of German students with SEN-L and the test fairness as compared to students attending basic secondary schools. Overall, these analyses revealed several promising findings: First, the number of missing values was rather low and, more importantly, did not differ between students with and without SEN-L. There was no indication that the instruction or the test material were too complex for students with SEN-L, thus, leading to response refusal or premature test termination. Second, the test exhibited a satisfactory model fit and represented an essentially unidimensional scale. Although the test was slightly too easy at the second measurement occasion, the items discriminated well between students with and without SEN-L. However, for a short instrument with few items some form of range restriction had to be expected as compared to full-fledged test batteries that are used in applied assessment. Third, although some items exhibited DIF between students attending special schools and regular schools, these effects were not systematic and did not affect mean-level comparisons between school types. Moreover, a closer inspection of these items did not reveal any specific features or underlying logical rules that might suggest a systematic bias for students with SEN-L. Therefore, the test allowed for a valid comparison of reasoning abilities between students with and without SEN-L. Finally, we also established partial longitudinal measurement invariance for students with SEN-L which allowed the examination of change trajectories in reasoning abilities. Overall, these results document satisfactory measurement properties of the reasoning test for students with SEN-L. The test not only allowed for longitudinal analyses but also fair comparisons with students from basic secondary schools.

It is notable that the present findings do not coincide with previous research that resulted in rather critical conclusions (e.g., Bolt and Ysseldyke, 2008; Südkamp et al., 2015). Domain-specific competence tests frequently showed poor measurement properties among students with SEN-L that did not allow for the estimation of valid person scores or comparative analyses with students from regular schools. These discrepancies might be explained by specific features of the administered test. The reasoning test was designed as an economical instrument that was rather short. The total testing time (including instruction) was at most 11 min (Lang et al., 2014). In contrast, competence tests in large-scale assessments are typically substantially longer. For the NAEP in the United States or the German NEPS students spend up to 1 h (or longer) on competence assessments. It is conceivable that students with SEN-L are unable to sustain their concentration for longer periods of time (Grünke and Grosche, 2014), particularly for challenging tests that require maximal performance. Moreover, the administered test had a rather simple design using, for example, a common multiple-choice response format for all items. In contrast, many competence tests adopt different, frequently rather complex, response formats (e.g., open responses, matching tasks; cf. Gehrer et al., 2013) that might increase the difficulty of the administered items (e.g., Hohensinn and Kubinger, 2009, 2011). Consequently, students with SEN-L might be inclined to engage in unsystematic random guessing to a larger degree rather than trying to solve a complex cognitive item (Wise and Kong, 2005) or even to omit these items rather than providing any response at all (Kato et al., 2007; Pohl et al., 2016). Therefore, the presented results should not be readily generalized to more complex achievements tests that are routinely administered in LSAs. Rather, our results highlight that it can be feasible of using a simple instrument that was originally designed for students from regular schools also for students with SEN-L.

### Limitations

The generalizability of our findings might be limited by some weaknesses of the presented study. For example, it is conceivable that some students from regular schools might have an undiagnosed SEN-L. Then, school tracks and SEN would not be perfectly redundant and, the reported DIF analyses would confound different effects to some degree. Moreover, our analyses focused on the internal structure of the reasoning test without evaluating its validity. We examined the dimensionality of the test and explored the fairness of the test for the comparison of students with and without SEN-L as well as for longitudinal comparisons within these groups. However, we did not scrutinize the criterion validity of the test in the different subgroups. Therefore, future studies should extend our findings by evaluating the predictive validity of the reasoning test, for example, to explain educational outcomes (e.g., grades) among students in special and regular school. Another limitation pertains to the different test modes adopted at the two measurement occasions. Whereas the first measurement administered paper-based tests, the second measurement used computerized tests. Although great care was taken to make the two assessments as comparable as possible, systematic mode effects cannot be ruled out (cf. Steger et al., 2018; Kroehne et al., 2019). For example, responding on the computer might be more difficult as compared to dealing with paper-based booklets because of differences in computer familiarity. As a result, the test mode might have distorted longitudinal comparisons to some degree. However, findings from previous mode experiments (Williams and McCord, 2006; Schroeders and Wilhelm, 2010) suggest that respective mode effects are likely to be negligible and no critical source of bias for reasoning tests such as the one administered in the present study. Nevertheless, future studies should explicitly evaluate whether test modes might have affected the measurement of reasoning abilities differently among students with and without SEN-L. Finally, we adopted a novel inference test to detect practically relevant DIF (Fischer et al., 2016). So far, little is known about the power of this test and how it fares in comparison to alternative methods for identifying non-negligible DIF (e.g., Wells et al., 2009; Casabianca and Lewis, 2018).

### Implications for LSAs

Educational large-scale assessments typically strive to gather a comprehensive picture of the student population in the respective country. Students with SENs pose a fundamental challenge for this goal because many standard instruments for the measurement of domain-general cognitive abilities or domain-specific competences have been shown to be unsuitable for these students. Consequently, cognitive assessments for students with SEN are frequently not available in many LSAs or simply not comparable to students from regular schools. Therefore, the present study evaluated a rather simple instrument for the assessment of basic reasoning abilities. In contrast to previous research on more complex cognitive tests such as reading competence (Südkamp et al., 2015; Pohl et al., 2016), our findings demonstrate that students with SEN-L can be incorporated into standard LSAs as long as the administered instruments are comparably simple. Our reasoning test included a common item format with a standard multiple-choice response scale that was easy to understand, even for disadvantaged students. Moreover, the test was rather short and did not require sustained attention over a long period of time. As a result, the test allowed for the estimation of valid proficiency scores that were comparable to students without SEN-L and even allowed for longitudinal comparisons over time. Overall, these results demonstrate the feasibility of integrating students with SENs into LSAs to assess basic cognitive abilities comparable to students from regular schools.

## Conclusion

Basic reasoning abilities can be validly measured in students with SEN-L using a short instrument for domain general cognitive functioning. The presented results demonstrate that the test was suitable for 16- and 22-year-old adolescents and young adults with SEN-L and allowed for comparative analyses with students from basic secondary schools as well as longitudinal analyses of developmental change over time. Because some items exhibited missing values and non-negligible DIF, researchers should adopt appropriate latent variable models to account for differences in the measurement structure of the test between school types and measurement occasions. Simple sum scores tend to underestimate between-group differences to some degree. Overall, these results demonstrate that students with SEN-L can be incorporated into educational large-scale assessments to measure their cognitive abilities comparable to regular students.

## Data Availability Statement

This paper uses data from the National Educational Panel Study in Germany. The anonymized data analyzed for this study will be available to the international research community free of charge at http://www.neps-data.de.

## Author Contributions

TG and LN wrote the manuscript. TG performed the statistical analyses.

## Conflict of Interest Statement

TG and LN are employed at the Leibniz Institute of Educational Trajectories that conducts the National Educational Panel Study. However, the institute had no involvement in the analyses of the data or the writing of the manuscript. The authors received no financial benefits for the publication of the manuscript.

## Footnotes

1 Students with a low academic performance but normal cognitive abilities are not diagnosed with a SEN-L (Grünke and Grosche, 2014).

2 A substantive research question of the study pertained to potential stigmatization and displacement effects of students with SEN-L. A comparison of students with similar cognitive profiles but different educational histories might help in identifying causal effects of specific schooling effects.

3 http://www.neps-data.de
